# Literature profiling on tourism, impairment and disability issues: A future directional guide

**DOI:** 10.4102/ajod.v11i0.862

**Published:** 2022-12-14

**Authors:** Tawanda Makuyana, Engelina du Plessis, Oliver Chikuta

**Affiliations:** 1 Department of Tourism Research Unit, Faculty of Economic and Management Sciences, North-West University, Potchefstroom, South Africa; 2 School of Tourism, Faculty of Economic and Management Sciences, North-West University, Potchefstroom, South Africa; 3 Department of Hospitality and Tourism, Faculty of Hospitality and Sustainable Tourism, Botho University, Gaborone, Botswana

**Keywords:** Inclusive tourism, access-needs in tourism, disability-impairment-tourism, disability-tourism research, future disability tourism research age

## Abstract

**Background:**

South African tourism is evolving towards accommodating disabled people. Within the same standpoint, the country receives ageing tourists as a major international tourism market from the Global North, whose access needs are similar to disabled people. The present article explored ‘blind and blank spots’ in the extant literature on tourism–impairment disability as a synchronised field within academic research to provide theoretical insights and gaps for the disability-tourism research community to consider the composite concept instead of individualistic concepts.

**Objective:**

The objectives were: (1) to track knowledge development from 1990 to 2018 using a narrative literature review approach and (2) to justify future research areas previously overlooked and understudied within a tourism–impairments–disability perspective in South Africa and beyond.

**Method:**

A narrative literature review search strategy was used. Keywords and synonymous terms were used in electronic searches of Scopus, ScienceDirect, Sabinet Online, Emerald Insights Journals, African Journals and Google Scholar. The literature screening process used predetermined inclusion and exclusion criteria for the data source. Content thematic analysis was adopted for the present study.

**Results:**

The findings reflect a dearth of tourism–impairments–disability research in South Africa. Nonetheless, there is an observable pattern of slow growth in research after the 2000s. The extant literature is skewed towards the tourism supply side and sporadic on tourism demand (tourist experiences), education and skills development.

**Conclusions:**

It is clear that the absence of scientifically developed knowledge on disability–impairments–tourism affects inclusive tourism growth. Therefore, the research community should consider disability-inclusive (accessible) tourism management, human resources and marketing practices and knowledge for teaching material in future research.

**Contribution:**

The article mapped and provided insights that sets a research agenda for tourism research community to see the gaps in literature and/or knowledge for accessible tourism (disability-inclusive) tourism to be a game changer as found by UNWTO (2020) with low-resources setting. Thereby setting a tone towards call for more research that can uncover an economic narrative that shows a relationship between skills development, labour and consumer markets for the participation of diverse disabled persons as such is shown as understudied in Low-to-Middle income earning countries like South Africa.

## Introduction

The Global North incepted activism and advocacy for disability inclusion through movements dating back to the 1970s (Nzo 2019). In the Global North, research and knowledge development in tourism began in the 1800s (Visser 2004). Nonetheless, tourism only began to be regarded in the academic research field in the 1970s in South Africa (La Fuente-Rodes et al. 2016; Adukaita et al. 2016; Booyens 2020). In the research space, the disability and tourism fields have been conducted separately in these past decades. This background draws attention to desktop research from 1990 to 2018, because it was a period: (1) covered by the primary study, of which the current article was an element and (2) when South Africa was positioned to be disability-inclusive through the Constitution of 1996, Chapter 2; the signing and ratification of the United Nations Convention for the Rights of Persons with Disabilities (UNCRPD) of 2006 in 2007; and the domestication of the said convention into a White Paper for Rights of Persons with Disabilities in 2015 (Kazou 2017). Despite the milestones mentioned above, disability, impairments (health and body conditions) and tourism as a joint field in research remained an area that was missing from the research agenda and the narratives and discourses (knowledge development) generated by scholars, universities (other higher education providers included) and research entities in South Africa.

According to Buhalis and Darcy (2013) and Darcy et al. (2020), impairment is the loss of limbs or parts of an individual body. On the one hand, disability has been an issue of debate amongst international, national organisations and individual academicians, of which researchers who are skewed towards medical models define disability as the inability of an individual to participate because of acquired impairment and health condition (Kazou 2017). On the other hand, disability researchers who uphold the social model’s perspective regard disability as more about the interaction between persons with impairments’ intrapersonal, interpersonal and environmental arrangements developed to solely cater to nondisabled counterparts (Vehmas & Watson 2014; 2016). Simbaya et al. (2019) have an all-around interpretation of disability as an evolving concept that aims to facilitate full, optimal and effective participation of a disabled person without compromising their health needs, dignity, choice and independence when participating any type of activities in any community.

The present article uses the term ‘disabled persons or disabled people’ to refer to individuals who acquired impairment, whether at birth or with life circumstances like ageing, incidents and accidents. The usage of the above mentioned term, disabled people, implies that unless there is universal accessibility, the said people are being obstructed by an environmental arrangement that exacerbates the loss or limitation of the body (Kazou 2017). The loss mentioned above is due to environments created by others that create physical or social barriers that disable or obstruct an individual with an impairment from participating equally with nondisabled people in the community’s normal life (Kazou 2017). Hence, the ‘ability’ of the individual who acquired an impairment is not being questioned, but the ‘disability’ can be associated with limited consideration for the access needs of the said people to participate in an environment that supports their capabilities (Buhalis & Darcy 2011). The argument in this article upholds the reality that both disabled and nondisabled people are heterogeneous in terms of their talents and role in socio-economic-based development. However, when it comes to empowerment initiatives like tourism education, it became a norm to bring access to information. Knowledge of diversity in all forms addresses attitudes, behaviours, choices, dignity, and independence of nondisabled people, whilst such is limited in considering disabled counterparts (Vehmas & Watson 2016; Simbaya et al. 2019; Makuyana & du Plessis 2022). As much as tourism’s co-created economy is concerned, there are misconceptions about disability and a lack of understanding of the impact caused by either impairment or health conditions on tourism growth (Makuyana 2020). The limited knowledge development in said field exacerbates misunderstanding of health conditions (acquired impairments) (Kazou 2017), talent and diversity (Gronvik 2007), the workplace (Groschl 2007; 2011) and tourism co-production and co-consumption (Darcy 2010). Therefore, the inclusion of disabled people is incomplete if socio-economic empowerment in tourism that upholds diversity is void of sufficient research that can inform the co-creation of resources to support inclusive tourism practices. For the study, tourism is considered a concept that describes the process when one voluntarily leaves one’s usual place of residence to travel to another environment for any purpose for less than a year (Camilleri 2018).

The current article identifies obstacles tourism practices face in education, community and industry because of the limited contextualised research-based knowledge beyond legal compliance (La Fuente-Rodes et al. 2016; Darcy 2010). In most tourism organisations within the value chain, disability inclusion is furthered by individuals who have disabled family members (Makuyana & du Plessis 2022). Thus, inclusion emerges more from personal than organisational practices (TraVability 2018; Tan 2014). The three concepts are always presented separately from the tourism co-created economy, which leaves tourism stakeholders in a dilemma regarding implementing disability inclusion in the day-to-day management and business operations (Shaw & Cole 2004; Tan 2014). Based in the South African tourism research context, if the above mentioned concepts maintain the discourse reflects:

- Tourism stakeholders’ misunderstanding of disability inclusion, results in challenges in accommodating the needs of disabled guests. (Snyman 2002).
- Mainstream tourism policy and other legislative frameworks (for education, community development and industry) are void of disability inclusion. Resulting in the formulation of separate disability policies that include disabled people in mainstream activities (Makuyana 2020).
- Tourism experiences amongst disabled tourists reflect systemic discriminative attitudes and behaviours when co-producing and co-consuming tourism products and services (Breedt 2007).
- Disability and/or accessible and/or inclusive tourism can fail to effectively grow as the market needs remain undiscovered (Department of Tourism report 2011; Makuyana & du Plessis 2020), and their expectations, motivations and needs remain unfulfilled (Chikuta et al. 2017; 2018).
- Tourism educators and industry roleplayers rely on intuitive know-how when managing their disabled counterparts. Such an approach embeds apathy and over-sympathy that can be disabling to disabled peers (La Fuente-Rodes et al. 2016; Makuyana & du Plessis 2022).

Disability inclusion has been an issue that remains in scholarly debates with a limited practical understanding of the concept among tourism role players resulting in South African tourism. (research, education, industry and hosting community included) ‘dragging its feet’. The Global North has identified accessible tourism as one of the main avenues for recovery after the coronavirus disease 2019 (COVID-19) pandemic (WTO 2020). Darcy et al. (2020) blazed a trail in the transformation of disability tourism from a niche, specialised or small market to an accessible, mature tourism market because:

- The Global North has more ageing tourists with adequate financial resources, supported with time to participate in tourism with more extended stays and spending, and they often travel with more than two people.
- Ageing tourists share similar needs with disabled tourists but don’t want to be labelled or identified as disabled tourists because of disablism that regards people who acquired impairments as homogeneous.

From a South African context, the Department of Tourism report (2011) shows only anectodical evidence on the readiness of the industry to handle accessible tourism because of a shortage of research on disability, impairment and tourism. If such existed, it would be easier for skills development to develop teaching material to prepare the public and private sectors for the realities of South African tourism. Thus, in the Department of Tourism report (2011), the commissioned researchers relied only on non-African (South Africa included) research to argue for accessible tourism. On the one hand, the present authors believe that a composite concept can alleviate the obstacles in knowledge development (research) to unveil the relationship between tourism, disability and impairment for tourism growth. On the other hand, a review of extant literature can identify research areas to allow the research community to consider future studies.

## Conceptual framework

**FIGURE 1:**
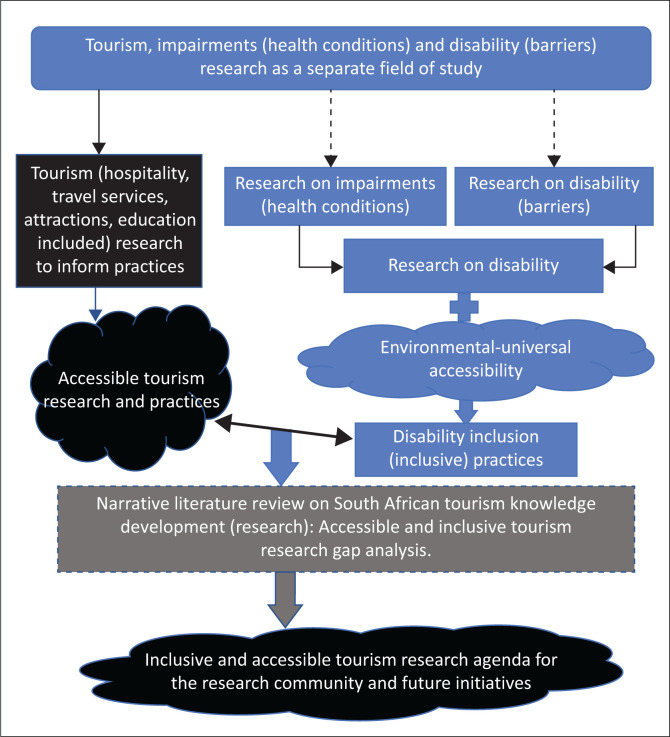
Conceptual framework for the study.

The current article presents a conceptual framework in Figure 1 below that shows tourism research has a parallel relationship to research on impairment and disability (exclusion and inclusion). The outcome and impact of research conducted as separate fields of study hardly relate to each other in principles and practice because of the absence of context and applicability.

Figure 1 shows that research on tourism (hospitality, travel services, attractions and education included) can have a reciprocal linkage with disability and impairment as a composite concept. Accessible tourism research would unveil disability-inclusive approaches that are rooted in knowledge development. The present authors believe tourism requires a body of knowledge that informs the reality of impairments (health conditions) and disability within tourism practices. Such scientific knowledge is critical to enhancing a comprehensive and balanced understanding of the concepts in a composite and the relationship thereof. Previous studies on impairments, disability and tourism are important to ascertain the status quo (gap analysis) whilst identifying areas towards greater understanding of this composite concept and giving direction for future research.

The objectives of the article are:

- to track knowledge development from 1990 to 2018 using a narrative literature review approach
- to justify future research areas previously overlooked and under-studied within a tourism–impairments–disability perspective in South Africa and beyond.

Hence, firstly, as Snyder (2019) recommended, a narrative literature search strategy is outlined under the methods and design that allowed the review processes to gather and then analyse data. Secondly, the results and findings are presented. Thirdly, the findings are discussed, and lastly, the conclusions are presented.

## Methods and design

### Context: South Africa

South Africa is in the G20 group from an economic and political position. The said group has countries in the Global North that have developed their systems towards inclusive approaches (Nzo 2019; also see Table 1 below). Hence, socio-economic and political-led migrations prevail amongst the countries in such groups (tourism comes into the picture) (Haarhoff & De Klerk 2019). Therefore, South Africa needs to conform to disability-inclusive tourism research (knowledge development). This is reinforced by Haarhoff & De Klerk (2019), who believe more than 66% of international tourism markets are tourists from Europe and the Americas who fall into the senior age category with access needs. In 2016, the tourists mentioned above spent R20 billion on leisure, accommodation and food in South Africa (Haarhoff & De Klerk 2019). Many international and local people amongst the 50+ age group have access needs, similar to disabled people. Yet usually, such people do not prefer to be ‘labelled or identified’ as disabled people.

**TABLE 1:** Examples of countries (SA and the international tourism market) that have pro-disability legal tools.

Countries	Year	Name of the legal frameworks
United States of America	1990	Americans with Disabilities Act of 1990
Zimbabwe	1992	Disabled Persons Act of 1992
Australia	1992	Disability Discrimination Act of 1992
United Kingdom	1996	Disability Discrimination Act of 1996
Republic of South Africa	1996 2007 2011 2016	Constitution of 1996, Chapter 2 Signed and ratified UNCRPD of 2006. National Development Plan vision 2030 White Paper for Rights of Persons with Disabilities

*Source:* Adapted and derived in part from Haarhoff, R. & De Klerk, B., 2019, ‘Destination South Africa: Analysis of destination awareness and image by international visitors’, *GeoJournal of Tourism and Geosites* 24(1), 201–211. https://doi.org/10.30892/gtg.24116-353

On the one hand, South Africa strives to adhere to such standards as the United Nations Convention for the Rights of Persons with Disabilities of 2006; the Sustainable Development Goals of 2015, which are supported by the Constitution of 1996; and other domestic legislative frameworks like the White Paper for the Rights of Persons with Disabilities of 2016 (see Table 1 below). Additionally, South Africa understands diversity in socio-economic settings (Meyer & Meyer 2020). Amongst the African countries, South Africa has indicated an interest in the accessible tourism market as a matter of business, legal compliance and social value and cohesion (Department of Tourism report 2011). The Department of Tourism has established a Transformation Committee with designated positions from the South Africa Disability Alliance. In addition, the Tourism Grading Council of South Africa has developed a disability inclusion framework for the accommodation sector in collaboration with the South Africa Disability Alliance. According to Haarhoff & De Klerk (2019), South Africa serves international markets from countries with pro-disability inclusion legislation, hence posing the need for research in tourism, impairment and disability, considered a composite field of study (see also Table 1 below).

Table 1 presents a list of examples of countries that are tourist source regions for South Africa and which have a legal position that advocates for disabled people. If South Africa relates to such states, the current authors believe that it is noble for South Africa to embed disability inclusion within tourism practices, thereby bringing the role of research to filling the research inconsistencies on impairments and disability issues within South African tourism and beyond. Therefore, the current study identifies areas previously researched and then provides information and/or gaps to enable the research community to conduct a more in-depth investigation and/or investigation on areas that have not yet been covered.

On the one hand, it is critical to take note of the relationship between impairments, disability and ageing, since approximately 53.0% of the global population live with a declared temporary and permanent impairment and have a propensity to travel (TraVability 2018; Disabled World 2016). On the other hand, global statistics record that 10% – 20% of the global population has a declared impairment (Darcy et al. 2020; United Nations Development Programme 2018). In South Africa, 7.5% of the South African population comprises the disabled population group (Statistics South Africa 2014). The point here is, generally, at one point in life, one can acquire a form of impairment due to life incidences such as ageing, short-sightedness, pregnancy, accidents or being born impaired, amongst other causes (Luiza 2010). However, the intensity and experiences of disability always differ. All the persons mentioned above share tourism access needs. However, the emphasis and the context of acceptance, discrimination, stereotyping and marginalisation by society and the co-created tourism economic environment may differ (Makuyana & du Plessis 2022; Kazou 2017). This implies that individuals experience diverse types of discrimination as per the societally and economically perceived level of the impairment whenever they participate in socio-economic activities like tourism (TraVability 2018; Disabled World 2016). The discussion above reinforces the need for research on the composite concept.

### Terms and Definitions

Disability is defined in this study as an interactive process between an individual with an impairment, the environment and the impairment at intrapersonal and interpersonal dimensions (Makuyana & du Plessis 2022). In this context, impairment is considered an act of God that brings about a loss or deformation of a ligament or body part (Chikuta 2015a; Makuyana & Saayman 2018). Access needs refer to the willingness and ability to be involved and/or participate in opportunities offered to all humans for tourism that upholds universal accessibility approaches (Makuyana & du Plessis 2022; Nzo 2019). Universal accessibility and/or disability inclusion refers to the inclusion of disabled people by any means which upholds one’s right to dignity, independence and choice to partake in all available opportunities or effective participation on an equal basis with nondisabled cohorts (Makuyana & du Plessis 2022; Darcy et al. 2020). Accessible tourism is the same as disability tourism and inclusive tourism, which implies the access needs of all people with a desire to participate in tourism regardless of the presence or absence of impairment. The terms mentioned above are used interchangeably, depending on geographical location (Darcy 2010; Buhalis & Darcy 2011).

### Design

The narrative literature review focused on the interconnection between tourism, impairments and disability from high-ranked databases that prefer developed countries (Global North) whilst taking cognisance of developing countries’ journals. The electronic search is done as a gap analysis that would provide answers to the following questions:

- What is the magnitude of research on tourism supply positioned in the context of inclusion of impairments and disability in South Africa and beyond?
- What lessons can be learned from existing research on tourism products and service development incorporating impairments and disability?
- To what extent does existing research embed tourism education (skills development) that incorporates impairments and disability for inclusive tourism growth?
- Is there research on the influence of technological developments on tourism, impairments and disability for effective participation amongst disabled people?
- Is there research (knowledge development) on impairment, disability and tourism as a combined concept for inclusive tourism growth in South Africa and beyond?

### Data collection

The researchers conducted a literature search from the following electronic databases: Scopus, ScienceDirect, Sabinet Online, Emerald Insights Journals, African Journals and Google Scholar. The electronic search used keywords like inclusive tourism, access-needs in tourism, disability, impairment, tourism and related synonymous terms. A balance of five regional (continental) journals with full text written in English were considered in the review. On the one hand, predetermined inclusion of sources of data entails published research work on tourism, impairment and disability issues. On the other hand, predetermined exclusion criteria entail nonacademic and scientific work on tourism, impairment and disability issues, as separate fields of study were considered during the literature screening process, as shown in Table 2.

**TABLE 2:** Review inclusion and exclusion criteria.

Inclusion	Exclusion
Keywords or synonymous terms	Articles that do not align with keywords or synonymous terms
Peer-reviewed, full text in English	Articles that are not peer-reviewed or in English
Peer-reviewed published reports and handbooks	Non-peer reviewed reports and handbooks
Academic work (e.g. books and theses on tourism impairments and disability as a composite concept)	Nonacademic and academic work on tourism, impairment and disability as separate fields of study
Country and continent used in the case study	N/A
Tourism main sectors and subsectors, e.g. hospitality (amenities, accommodation, food and beverages), natural and man-made attractions (parks, theme parks, recreational parks, travel services)	Non-tourism sectors
Timeframe of the published articles (1990-2018)	Timeframe before 1990 and after 2018
Research focus and themes aligned with impairments, disability and tourism under the following: Tourism demand side Tourism products and service development Tourism supply side Tourism education and skills development Generic and general tourism Technological developments	Disability and impairments and tourism research areas as separate fields of study

Note: The aim is to track knowledge development from 1990 to 2018 whilst justifying future research areas which would have been previously overlooked and understudied within a tourism–impairments–disability perspective in South Africa and beyond.

Table 2 shows an objective electronic literature search guide as part of the reviewing process. The current article is an element of the unpublished primary (PhD) study by Makuyana (2020). The review paper would be published for the tourism, disability (academics and scholars) research community, practitioners and general readership to have access to the information beyond being kept in the university’s repository.

### Data analysis

**FIGURE 2:**
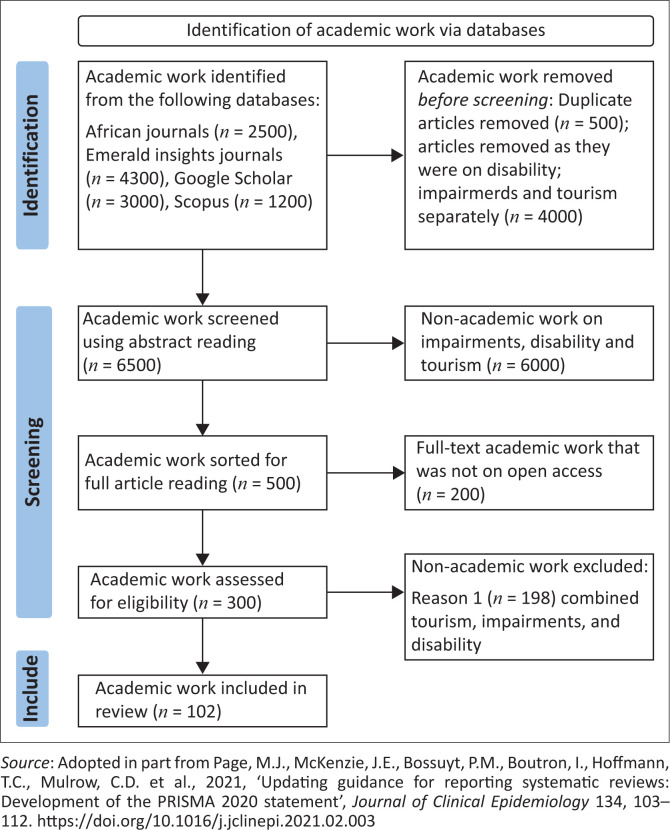
Summary of data collection, screening and analysis process.

The first and second authors independently reviewed each identified data source to determine eligibility and extract study information. The third author validated and verified the collected data whilst preparing and refining the article to make it ready for publication. Figure 2 shows the volume of studies identified, screened and included or excluded at each stage of study selection. All collected data were categorised into themes answering the research questions, as advised by Miles and Huberman’s (1994) qualitative analysis approach. Figure 2 shows that the researchers retrieved data and adopted three steps. Firstly, an electronic literature search and second screening of research material from the 1990s to 2018 was conducted. The researchers followed Vergnes et al. (2010) by grouping the codes into themes, which established a scientific way of synthesising a plethora of information after exhaustively searching and objectively analysing (reduction and exploration of text) the studies dealing with tourism–impairments–disability issues. As reflected in Figure 2, research work in text format was gathered after reading the titles and abstracts. Full-text reading ascertained the alignment with the aim of the current review. All materials which addressed tourism, disability and impairments separately and work that was not on open access were discarded.

Secondly, data were extracted, and thirdly, data were analysed manually following a thematic approach as advised by Miles and Huberman (1994), and in vivo coding was also carried out (Holton 2007). The questions calling for narratives were subject to inductive or deductive thematic analysis, using in vivo open coding as Manning (2017) advised.

Examples of questions subject to inductive analysis:

- What is the magnitude of research on tourism supply positioned in the context of inclusion of impairments and disability in South Africa and beyond?
- What lessons can be learned from existing tourism research on products and service development that has incorporated impairments and disability?

Examples of questions subject to deductive analysis:

- To what extent does existing research embed tourism education (skills development) that incorporates impairments and disability for inclusive tourism growth?
- Is there research on the influence of technological developments on tourism, impairments and disability for effective participation amongst disabled people?
- Is there research (knowledge development) on impairment, disability and tourism as a combined concept for inclusive tourism growth in South Africa and beyond?

This technique involves meaningful units of texts or codes being extracted, kept in a master list and reapplied to similar segments of text. Codes were also transformed into dummy variables in Microsoft Excel (for each code: 0 = not mentioned, 1 = mentioned in the extant literature) to represent code frequencies illustrated in numeric tables and percentages (quasi-statistics).

## Results

The article aims to track knowledge development from 1990 to 2018 whilst justifying future research areas which would have been previously overlooked and under-studied within a tourism–impairments–disability perspective in South Africa and beyond. The findings are presented first to show the geographical or geospatial distribution from a global scope before narrowing it to South African-focused knowledge development. This is because of the interconnection of tourism co-created supply, demand and internationalisation of research and skills development.

### Data distribution within the electronic databases (sources)

A total of 300 articles were reviewed, and only 102 articles from the four databases were used. The databases enabled access to 18 tourism and/or hospitality-related journals that had 55 articles and 11 journals unrelated to tourism–impairments–disability that had 40 articles. These were augmented by one tourism encyclopedia, three European and international organisational reports and one university repository that had two PhD theses, two master’s dissertations and one handbook, as shown in Table 3.

**TABLE 3:** Sources of data used for this study (in alphabetical order).

Names of sources of data	Total resources used
African Journal of Disability	2
African Journal of Hospitality, Tourism and Leisure	5
Annals of Faculty of Economics	2
Annals of Tourism Research	10
Channel View Publications	8
Cornell Hotel and Restaurant Administration Quarterly	6
Cross-Cultural: An International Journal	2
Disability and Rehabilitation	2
Encyclopedia of Hospitality and Tourism	1
Euromontana Communication Tourism Report	1
Europe for All Report	3
European Commission Report	1
Hospitality Management	2
International Cases in Tourism Management	1
International Journal of Contemporary Hospitality Management	4
International Journal of Hospitality Management	3
International Journal of Tourism Research	2
International Journal of Hospitality and Tourism Administration	2
Journal of Hospitality and Tourism Management	5
Journal of Sustainable Tourism	1
Journal of Tourism Future	3
Journal of Travel Research	1
Manly Pacific Conference proceedings	2
New South Wales Ageing and Disability Department	1
North West University -Repository	4
OSSATE	5
Review of Disability Studies	4
STCRC technical report	1
Tourism and Hospitality Research	2
Tourism Management	12
Tourism Planning and Development	1
Tourism Review international	1
TRANSCEND	1
University of Technology Sydney Institute for Public Policy and Governance	1

**Total**	**102**

Table 3 records the distribution of tourism, disability and impairments as disaggregated by subject, namely tourism–impairments–disability, from 34 sources. However, the research revealed that scientific tourism-related journals have not yet regarded the tripod concept, namely tourism–impairments–disability (where impairments include health conditions), as one of their central research foci or themes. Tourism and hospitality sources contributed 51% of the data, whilst non-disability-related sources contributed 34%. A university repository and encyclopedias on tourism were 3%, whilst European organisational reports provided nine percent 9%. Table 2 highlighted the 44% (general percentage distribution) of tourism, disability and impairment literature data relevant for this analysis and reviews: 11.7% of the articles were published through the *Tourism Management Journal*, followed by *Annals of Tourism Channel View Publications* publishing 7.8% of the articles and *Cornell Hotel and Restaurant Administration Quarterly* publishing 5.9% of the articles, whilst 4.9% were published by *African Journal of Hospitality, Tourism and Leisure, Journal of Hospitality, Tourism Management* and OSSATE, respectively; 3.9% were published by *Review of Disability Studies*, the North-West University repository and *International Journal of Contemporary Management*, respectively.

### Identified themes and subthemes

The analysis established five themes, namely:

- tourism demand: disabled people (tourists)
- tourism supply: inclusion of disabled people (tourists), impairments and health conditions and disability
- technological influence on tourism, impairments and disability
- education and skills development
- tourism-inclusive practices (generic knowledge and theoretical and literature base).

The themes had subthemes as follows:

- Tourism supply had seven subthemes, namely heterogeneity, inclusion, reasonable accommodation, know-how, managing experiences during co-consumptions and co-production and product and service innovation.
- *Tourism demand: disabled people (tourists)* had three subthemes: escapism, relaxation and recreation. *Education and skills development* had two subthemes, namely empowerment and self-development. *Tourism-inclusive practices (generic tourism knowledg*e) had subthemes like involvement and participation.

#### Tourism demand: Disabled people (tourists)

The results are clustered under tourism demand, impairment and disability. Table 3 and Table 4 summarise the knowledge distribution by country and continent where the study was conducted. From the view of tourism demand, five studies covered the whole of Europe, including Spain, Poland and the Czech Republic. Six studies focused on the Asia-Pacific region, including studies that focused explicitly on Taiwan, South Korea, India and China, whilst others focused on the United Kingdom and Middle East (Jordan), respectively. Two studies regarded Australia, both Americas and Africa (South Africa) individually.

**TABLE 4:** Research on tourism demand, impairments and disability and disabled tourists (in sequential order of years, from 1997 to 2018).

Country or Continent	Number of Authors	Authors	Year	Sector	Theme (area of focus)	Methodology and design
Europe in General	1	Report for European Union	1997	Generic tourism industry	Making Europe more accessible	Qualitative
Asia-Pacific	1	Conference report for ESCAP	2000	Generic tourism industry	Barrier-free tourism for people with disabilities	Qualitative
United Kingdom	2	Burnett & Baker	2001	Travel	Assessing the travel-related behaviours of the mobility-disabled consumer.	Qualitative
Australia	2	Yan, McKercher & Packer	2004	Generic tourism industry	Tourism from a determinants-constraints view within the demand side.	Qualitative
Taiwan	2	Chang & Chen	2011	Travel	Identifying mobility services needs for disabled air passengers	Qualitative
America	3	Shi, Cole & Chancellor	2012	Leisure tourism	Understanding leisure travel motivations of travellers with acquired mobility impairments.	Qualitative
Taiwan	2	Chang & Chen	2012	Travel	Factors that influence meeting the needs of disabled air passengers	Quantitative
South Korean	3	Lee, Agarwal & Kim	2012	Travel	Influence of travel constraints on intentions of travellers with disabilities to travel.	Qualitative
Jordan	1	Allan	2013	Tourism activities	Reasons persons with impairments engage in tourism activities.	Qualitative
America	2	Kim & Lehto	2013	Generic tourism industry	Establishing the motives and activities of families travelling with children possessing disabilities.	Qualitative
Spain	3	Navarro, Andreu & Cervera	2014	Accommodation	Value co-creation amongst hotels and customers with disabilities using an exploratory approach	Mixed methods
Spain and Australia	3	Dominguez Vila, Darcy & Alen	2015	Generic tourism industry	Competing for the disability tourism market: A comparative exploration of the factors of accessible tourism competitiveness in Spain and Australia	Mixed methods
Indian	2	Bindu & Devi	2016	Generic tourism industry	Accessible tourism: Determinants and constraints from a demand-side view.	Quantitative
Poland	1	Popiel	2016	Generic tourism industry	Tourism market, disability and inequality from a problem-solution perspective.	Qualitative
Czech Republic and Poland	1	Valkova	2016	Generic tourism industry	Challenges of persons with impairments in tourism for all approaches.	Qualitative
China	2	Kong & Loi	2017	Generic tourism industry	Barriers to holiday-taking for visually impaired tourists and their families	Qualitative
South Africa and Zimbabwe	3	Chikuta, du Plessis & Saayman	2017	Attractions	The motivations of tourists with disabilities as aligned with nature-based tourism	Mixed methods
South Africa and Zimbabwe	3	Chikuta, du Plessis & Saayman	2018	Attractions	The expectations of tourists with disabilities in National Parks	Mixed methods

**Total as at 2018**	**37**	-	-	-	-	-

Table 4 indicates available knowledge in the type of collaborations amongst authors. On the one hand, seven studies were conducted by two researchers (co-authoring), whilst others were conducted by three researchers (co-authoring). On the other hand, five studies were undertaken by single authors. Table 4 shows Taiwan and South Africa had authors who each undertook two related studies on this subject, respectively. The rest of the studies were one-off investigations into disability, implying that there is low interest in studying disability and tourism as a combination of two fields.

Table 4 shows 1997 as the genesis of research interest in disability and tourism demand in Europe from a generic European perspective. Table 4 indicates a break in research from 2000 to 2001, when the tourism and disability agenda was raised from a demand perspective. It presents 2006 as a year when research on tourism demand and disability resumed after further research silence. According to Table 4, a five year silence lasted until 2011. Table 4 shows 2011 to 2018 as having an exponential positive interest in developing knowledge on tourism (including attractions and activities), hospitality (accommodation) and disability and disabled persons as combined fields.

According to Table 4 above, between 1997 and 2000, research was skewed towards the subtheme of inclusion (universal accessibility) of disabled people (ageing people included) in tourism. In 2001, the research considered barrier-free (disabling environment alleviated) tourism for disabled people. In the same year, behaviours of tourism consumers with mobility impairments were documented. Table 4 presents 2006 as the year researchers identified determinants and constraints within the tourism demand side. Table 4 shows 2011 as the year that witnessed a widened focus to include identifying the needs, motivations and expectations of disabled tourists.

According to Table 4, 44% of the literature concentrated on barriers and challenges and constraints faced by persons with different impairments. Twenty-two percent of the demand-skewed research focused on the motivations of persons with impairments (disability). Seventeen percent of studies concentrated on persons with impairments’ needs and expectations. Competitiveness of accessible destinations, the role of persons with different impairments in co-creation and consumer behaviour received 6% of the research attention, respectively. Finally, Table 4 presents a distribution of methods of research, which was skewed towards qualitative methods with 67%, a quantitative research approach with 11% and mixed methods with 22%.

#### Tourism supply: Inclusion of disabled people, impairments (health conditions) and disability

The research positioning the tourism supply side in relation to disability and impairments knowledge (research) as of 2018 is presented in Table 5 below.

**TABLE 5:** The tourism supply: inclusion of disability, impairments and disabled people in sequential order of years from 1990 to 2018.

Country and region	Number of authors	Authors	Year	Sector	Theme (area of focus)	Design
Australia (Oceania)	1	Darcy	2000	Generic tourism industry	Tourism industry supply-side perceptions of providing goods and services for people with disabilities	Mixed methods
Generic (global tourism)	2	Kay & Russette	2000	Accommodation	Hospitality management competencies	Quantitative
Australia (Oceania)	1	Darcy	2002	Tourism	Marginalised participation of physical disability, high support needs and tourism	Mixed methods
Asia	2	Chi & Qu	2003	Food and beverage	Integrating persons with disabilities into the workplace: A study on the employment of people with disabilities in the food service industry	Mixed methods
Generic (global tourism)	4	McKercher, Packer, Yau & Lam	2003	Travel	Travel agents as facilitators or inhibitors of travel: Perceptions of people with disabilities	Qualitative
United Kingdom	2	Shaw & Coles	2004	Generic tourism industry	Disability, holiday-making and the tourism industry in the UK: A preliminary survey	Qualitative
Canada (America)	1	Groschl	2005	Accommodation and hotel sector	Persons with disabilities: A source of nontraditional labour for Canada’s hotel industry	Mixed methods
Generic (global tourism)	3	Daniel, Rodgers & Wiggins	2005	Travel	‘Travel Tales’: An interpretive analysis of constraints and negotiations to pleasure travel as experienced by persons with disabilities	Qualitative
Generic (global tourism)	3	Slonaker, Wendt & Baker	2007	Food and beverage	Employment discrimination in the restaurant industry	Quantitative
Canada (America)	1	Groschl	2007b	Accommodation	An exploration of HR policies and practices affecting the integration of persons with disabilities in the hotel industry in major Canadian tourism	Qualitative
Turkey (Europe)	3	Ozturk, Yayli & Yesiltas	2008	Accommodation and travel	A hotel and travel managers’ evaluation of the readiness of the Turkish tourism industry for the disabled (customer) market	Qualitative
Australia (Oceania)	1	Department of Economic Development, Tourism and Arts	Not provided	Generic tourism industry	Accessible resource kit	Qualitative
United Kingdom	3	Richards, Pritchards & Morgan	2010	Generic tourism industry	National report on a survey on accessibility provision	Mixed methods
Australia (Oceania)	1	Darcy	2010	Travel	Challenges in travelling as scoping travel medicine and disabled travellers	Mixed methods
Generic (global tourism)	1	Luiza	2010	Generic tourism industry	Ignored opportunities in accessible tourism	Mixed methods
Canada (America)	1	Groschl	2011	Accommodation	Diversity management strategies of global hotel groups: A corporate website-based exploration.	Qualitative
Denmark (Europe)	2	Blichfeldt & Nicolaisen	2011	Generic tourism industry	Disabled travel is not easy but doable	Qualitative
Spain (Europe)	1	Pagan	2012	Generic tourism industry	Time allocation for persons with disabilities in the tourism	Quantitative
Portugal (Europe)	3	Figueiredo, Eusebio & Kastenholz	2012	Leisure and hospitality	How diverse are tourists with disabilities? A pilot study on accessibility leisure tourism experiences in Portugal	Quantitative
Asia	3	Ju, Zhang & Pacha	2012	Generic tourism industry	Employers value employability skills as essential for entry-level employees with and without disabilities	Quantitative
Generic (global tourism)	2	Jasper & Waldhart	2012	Leisure and hospitality	Employer attitude towards hiring employees with disabilities in the leisure and hospitality industry	Qualitative
Generic (global tourism)	2	Kim & Letho	2013	Generic tourism industry	Travel by families with children possessing disabilities: Motives and activities	Mixed methods
Europe	1	Euromontana Communication Tourism	2014	Generic tourism industry	Accessible tourism in Europe: An attractive economic market, including mountain destination	Qualitative
Europe	1	Bowtell	2015	Travel and leisure	Assessing the value and market attractiveness of the accessible tourism industry in Europe: a focus on significant travel and leisure companies	Mixed methods
Australia (Oceania)	3	Vila, Darcy & Gonzalez	2015	Generic tourism industry	Competing for the disability tourism market from a comparative factor analysis	Mixed methods
England (United Kingdom)	1	Visit England	2015	Generic tourism industry	Re-envisioning tourism and visual impairments	Qualitative
Generic (global tourism)	1	UNWTO publication (report)	2015	Generic tourism industry	Manual on accessible tourism for all: Good practices in the public and private sector	Qualitative
Zimbabwe (Africa)	1	Chikuta	2015b	Generic tourism industry	Incorporating persons with disabilities in tourism planning	Qualitative
Poland (Europe)	1	Zajadacz	2015	Generic tourism industry	The contribution to the geography of disability to the development of accessible tourism	Mixed methods
Generic (global tourism)	1	UNWTO publication (report)	2016	Generic tourism industry	Manual on accessibility tourism for all (principles, tools and best practices)	Qualitative
China (Asia)	2	Loi & Hong	2017	Generic tourism industry	People with disabilities in the tourism industry (concept and issues)	Qualitative
Zimbabwe (Africa)	1	Chikuta, Du Plessis & Saayman	2017	Accommodation	The absence of disability issues in the hotel policy in Zimbabwe	Qualitative
Germany (Europe)	1	Rebstock	2017	Travel	Economic benefits of improved accessibility to transport systems for the role of transport in fostering tourism for all	Quantitative
England (United Kingdom)	1	Visit England	2018a	Generic tourism industry	Listen up to persons with hearing impairments	Qualitative
England (United Kingdom)	1	Visit England	2018b	Generic tourism industry	Accessibility non-participation	Qualitative

**Total as at 2018**	**59**	-	-	-	-	-

Table 5 shows 35 research items, revealing country and continental distribution of tourism supply-oriented research that considered disability and impairment issues as: (1) Australia (Oceania) accounts for 17%; (2) Asia in general accounts for 9%; (3) the United Kingdom make up 14%; (4) Canada accounts for nine percent 9%; (5) Turkey, Denmark, Spain, Portugal and Poland plus Europe in general have a combined aggregate of 23%; (6) Zimbabwe accounts for three percent 3%; and (7) Global tourism in general adds up to 26%. Table 5 shows a limited (if not a lack of) interest in tourism supply and disability until 2000. Uninterrupted growth in research output was witnessed after 2005, although 2006 and 2007 present a break in research in this regard. However, there was consistent research on tourism supply and disability from 2007 and 2008 until 2018.

Table 5 reflects the distribution of tourism supply and disability research; however, it is dominated by 57% of single authorship. Articles with two and three co-authors have 20% each, respectively, whilst articles with four authors have 3% only. Thus, authors like Darcy, Groschl, Chikuta, Visit England and United Nations World Tourism Organization (UNWTO) have recurring tourism and disability-oriented research from the supply-side perspective (see Table 5). The findings reflect tourism and disability studies within the supply-side as considering ageing as one of the major tourism market segments. This concurs with the World Travel Tourism Council report (2017) that highlighted that amongst the international visitors for the past decade, more than sixty-six percent (66%) fall into a senior market that prefers accessibility in co-producing and/or co-consuming tourism products and services as the disabled people. The above discussion is in the context of the growth of accessible tourism which scholars regard as having three times the growth rate of disability-exclusive tourism. This is because of the ageing and retiring baby boomer generation, which dominates the demographics of most of the Global North (TravAbility 2020). TravAbility (2020) cites research by McKinsey and Company in the United States of America (USA) that found that the baby boomer generation controls 60% of total wealth, and 40% of their total expenditure is in areas such as hospitality and leisure amongst 50% of the population segment. In addition, the said population segment, at age 65, has 40% of the cohorts with an age-related disability, and by age 75, it rises to 60%. It is apparent that the ones without declared impairments and the support structures of the counterparts with declared impairments prefer the usage of universal accessible facilities foregoing exclusive ones (TravAbility 2018). Such travel characteristics are observable among the majority if visitors to South Africa as a tourism destination (Makuyana & du Plessis 2020).

The studies on tourism supply and disability were undertaken between 1990 and 2018 and considered a variety of tourism sectors: the general tourism industry had 57.0%, the accommodation sector only had 14.0%, the hospitality sector (accommodation and hotels) had 2.9%, tourism only had 2.9%, food and beverage had 5.7%, travel only had eight comma six percent 8.6%, leisure and accommodation had 2.9%, accommodation and travel had 2.9% and travel and leisure had 2.9%. An interesting aspect highlighted in Table 5 is that the year 2000 ignited greater progressive research on access needs (disability) of the tourism market and supply chain.

#### Tourism supply: Disability inclusion and impairment issues in products and services development

Table 6 reveals that knowledge of disability and tourism can contribute to man-made and natural tourism products and service development and innovation that embraces the diverse needs of tourism market segments. Within this context, the current researchers classified such literature under research aligned with the tourism products and/or services from 1990 to 2018, which is presented in Table 6.

**TABLE 6:** Research on tourism products and services that accommodates disabled people (in sequential order of years).

Country or Continent	Number of authors	Authors	Year	Sector	Theme (area of focus)	Design
South Africa	1	Snyman	2002	Generic tourism industry	The needs of tourists with disabilities	Qualitative
United Kingdom	2	Shaw & Cole	2004	Generic tourism industry	Disability as contextualised in holiday-makers and the tourism industry in the UK	Qualitative
Australia	1	Darcy	2006	Travel	Experiences of persons with disabilities as aligned to the air travel chain	Qualitative
Australia	1	Darcy	2007	Accommodation	A methodology for assessing class three accessible accommodation- information provision	Qualitative
United States of America (USA)	2	Grady & Ohlin	2009	Accommodation	Equal access to hospitality services for guests with mobility impairments under the Americans with Disabilities Act: Implications for the hospitality industry	Qualitative
Australia	1	Darcy	2010	Tourism and Accommodation	Inherent complexity: Disability, accessible tourism and accommodation information preferences	Qualitative
Australia	3	Small, Darcy & Packer	2012	Generic tourism industry	The embodied tourist experience of people with vision impairments from a management angle: Setting a research agenda for Accessible Tourism	Qualitative
Portugal	1	Figueiredo, Eusebio & Kastenholz	2012	Leisure tourism	Diversity amongst tourists with disabilities as a pilot study for accessible leisure tourism experiences in Portugal	Qualitative
USA and the Republic of Korea	3	Kim, Stonesifer & Han	2012	Accommodation	Accommodating the disabled hotel guest: Implications for the hotel management and guests	Qualitative
South Africa and Zimbabwe	1	Chikuta	2015	Attractions	Universal accessibility in parks (unless referring to specific parks), e.g. SAN Parks	Mixed-Method
United Kingdom	1	n/a	2016	Leisure tourism	Reasons for making beer pubs accessible volume and value of accessible tourism in England	Qualitative
South Korea (Republic of Korea)	1	Lyu	2017	Tourism and accommodation	Tourism products, which people with disabilities are willing to pay more for	Qualitative
Turkey	1	Tütüncü	2017	Accommodation	Accessibility factors in hotels bolster the satisfaction of people with physical disabilities	Qualitative
United Kingdom	1	The National Autistic Society	2018	Events tourism	Welcoming autistic people by guiding them at tourist venues in England will increase the number of tourists	Qualitative

**Totals as at 2018**	**20**	-	-	-	-	-

n/a, not applicable.

Table 6 presents 2002 as the landmark year for tourism research that focused on the needs of disabled people from a tourism and hospitality product and service development perspective. According to Table 6, a two year research gap appeared until 2004; another 2 years of research silence was observed between 2004 and 2006. This was followed by another one year research break between 2009 and 2010, and then a 2-year gap occurred again between 2010 and 2012 (see also Table 6). Table 6 presents a three year research gap. Nevertheless 2015 had a consistent flow of research until 2018.

Table 6 presents 21% of the research themes skewed towards tourism product development. Whilst 29% of studies within the said period focused on experiences of the disabled tourist during co-production and co-consumption of tourism products, only 7% of the studies established a nexus between disability and tourism products and services. Within this tourism product or service-centred research, accessibility to information had 14%, whilst universal accessibility received 29% of research attention.

Table 6 shows the inclusion of disabled people within the different types of tourism services and products in different continents and countries. Thus, according to Table 6, Oceania had 40% of the research output, whilst Europe had 33%. Table 6 presents America and Africa with 13% research attention, respectively. Within this continental view, 36% of the research used Australia as a case study, 7% adapted America, 21% of research used the United Kingdom and Europe and Africa contributed 14% of the case studies, respectively.

#### Technological influence on tourism, impairments and disability

The results revealed that tourism, like any other sector, has been continuously affected by the Internet of Things, amongst other technological advancements that enhance business efficacies if harnessed strategically. Thus, as highlighted in Table 7, disability-tourism studies reflect either the adoption or proposition of technological innovations, communications (marketing and public relations included) and intrapreneurial and entrepreneurial approaches that can take advantage of technology for a wider reach to disabled people. These can enable them to have better access to participate in tourism and hospitality.

**TABLE 7:** Research on the technology development of disability tourism (in sequential order of year, from 1990 to 2018).

Country or continent	Number of authors	Authors	Year	Sector	Theme (area of focus)	Design
Europe	4	Buhalis, Eichhorn, Michopoulou & Miller	2005	Generic tourism industry	Accessibility market and stakeholder analysis: one-stop-shop for accessible tourism in europe (OSSATE)	Qualitative
Europe	4	Buhalis, Michopoulou, Michailidis & Ambrose	2005	Generic tourism industry	Developing a one-stop-shop for accessible tourism in Europe (OSSATE Portal) for the disabled tourism market: Technical and business challenges	Qualitative
Europe	4	Buhalis, Michopoulou, Ambrose, & Michailidis	2006	Generic tourism industry	An e-Tourism portal for the disabled tourism market in Europe: The OSSATE portal design (one-stop-shop for accessible tourism)	Qualitative
General	4	Eichhorn, Miller, Michopoulou & Buhalis	2008	Generic tourism industry	Enabling access to tourism through information schemes	Qualitative
General Europe	3	Darcy, Michopoulou, Ambrose & Buhalis	2011	Generic tourism industry	Special issues editorial on accessibility tourism in future	Qualitative
Australia (Oceania)	1	Darcy	2012	Tourism and accommodation industry	Disability framework for the action: Inherent complexity in disability, accessible tourism and accommodation information preferences	Qualitative

**Total as at 2018**	**20**	-	-	-	-	-

Table 7 shows a trend in studies that focused more on the continental level than on the country. According to Table 7, studies from 2005 to 2012 were 83% focused on Europe and 17% on Australia. Nonetheless, Table 7 shows a research gap in the nexus of tourism and disability influenced by technological development from 1990 to 2005. However, as mentioned earlier, research interest was reignited in 2005 and 2006, with a break between 2006 and 2008 (see Table 7). A research gap was observed from 2008 to 2011, followed by two years of continuous research between 2011 and 2012. After 2012, there was research silence on technological development in tourism and disability until 2018. The results reveal an interesting aspect of the exponential growth of research on technological advancements for tourism and disability, followed by research silence. On the one hand, a trend towards a collaborative research approach is preferred to single authorship, and only one solo author conducted research in the period under investigation. Table 6 shows that all studies adopted a qualitative research design.

#### Tourism education (skills development) on impairments and disability (inclusion)

Table 8 presents the findings that tourism competencies and/or education incorporate disability. According to Table 8, from 1990, there has been a shortage of tourism education or skills development research oriented towards health conditions or acquired impairments and disability inclusion from an Afrocentric perspective. Nonetheless, it is not until 2018 that South Africa (Africa) recorded 20%, Slovenia (Europe) recorded 60% and the world in general recorded 20%. Table 8 fosters the view that skills development informs and enhances know-how to prepare learners to manage and engage disabled counterparts in tourism.

**TABLE 8:** Research on tourism education and/or skills development, impairments and disability.

Country or region	Number of authors	Authors	Year	Design	Sector	Theme (area of focus)
General	2	Sigala & Baum	2003	Qualitative	Generic tourism industry	Trends and issues in tourism and hospitality higher education: Visioning the future
Slovenia (Europe)	3	Bizjak, Knezevic & Cvetreznik	2010	Qualitative	Generic tourism industry	A tourism student’s perspective on attitude change towards guests with disabilities
Slovenia (Europe)	3	Bizjak, Knezˇevic & Cvetrezˇnik	2011	Qualitative	Generic tourism industry	Attitude change towards guests with disabilities: Reflections from tourism students.
Europe	N/A	European Commission, Final Report	2014	Qualitative	Generic tourism industry	Mapping skills and training needs to improve accessibility in tourism services
South Africa (Africa)	2	Makuyana & Saayman	2018	Qualitative	Generic tourism industry	The postulate for the systematic mainstreaming of impairments in tourism education in South Africa: A literature synthesis

**Total as at 2018**	**10**	-	-	-	-	-

Table 8 reveals a preference for co-authoring amongst the researchers, as in previous themes. It is observed that after 2003, there was a seven year research silence on tourism skills development relating to disability for the tourism value chain in general (see Table 8). In 2010 and 2011, the same authors investigated attitude changes of students undergoing tourism skills development towards disabled guests. Such research reflects co-creation that places the role played by formal skills development as critical in the co-production and co-consumption of tourism and hospitality practices. Table 8 presents a three year research silence between 2011 and 2014. However 2014 broke the silence for a moment, and the aftermath witnessed a research break again until 2018 (see Table 8).

Table 8 highlights that Europe is making strides in disability and/or accessible tourism-related skills development or education research. Nonetheless, there is still a need to develop more integrated tourism–impairments–disability knowledge within the contexts of individual countries (see Table 8). On the one hand, Table 8 shows limited research attention on tourism, impairments and disability as a composite concept within the formal human capital-capacity development in Africa, especially South Africa. Table 8 reflects implications that can emerge from the lack of professional capacity to manage and/or handle learners with impairments amongst tourism educators (Makuyana & du Plessis 2022; Makuyana & Saayman 2018; Scott, McGuire & Shaw 2003). It goes without saying that the said gap fosters challenges in the capacity and capability of the tourism and hospitality roleplayers (practitioners) when serving disabled tourists (guests) within the tourism value chain (Breedt 2007; Chikuta 2015a; Makuyana & du Plessis 2022; Snyman 2002; see also Table 8). The findings implicate the tourism and hospitality workplace readiness and know-how on disability inclusion as part of diversity management practices.

#### The research on general tourism, disability and impairments issues

Table 9 presents generic tourism, disability and impairments research within the tourism business system that thrives as a co-creation economy.

**TABLE 9:** Results of general tourism literature on disability and impairment issues (from 1990–2018).

Country or region	Number of authors	Authors	Year	Sector	Theme (area of focus)	Design
General	2	Ray & Ryder	2003	Travel	‘‘Ebilities’’ tourism: An exploratory discussion of the travel needs and motivations of the mobility-disabled	Quantitative
General	3	Yau, McKercher & Packer	2004	Travel	Travelling with a disability: More than an access issue	Qualitative
General	2	Horner & Swarbrooke	2004	Tourism and travel	Tourism and travellers with disabilities	Qualitative
Brazil	1	Rains	2004	Travel and hospitality	Universal design and the international travel & hospitality industry	Qualitative
General	1	Ross	2004	Tourism and hospitality	Ethics, trust and expectations regarding the treatment of disabled staff within a tourism/hospitality industry context	Qualitative
General	1	Buhalis, Eichhorn, Michopoulou & Miller	2005	Tourism	Accessible market and stakeholder analysis	Qualitative
General	1	Dwyer	2005	Tourism	Relevance of triple bottom line reporting to the achievement of sustainable tourism: a scoping study	Quantitative
Australia	1	Darcy	2006	Tourism	Setting a research agenda for accessible tourism	Qualitative
Canada	1	Groschl	2007a	Hospitality	Cultural diversity in hospitality work	Qualitative
Europe	n/a	Europe For All	2007a	Travel	Europe for All – Better information for discerning travellers	Qualitative
General	3	Packer, McKercher & Yau	2007	Tourism	Understanding the complex interplay between tourism, disability and environmental contexts	Qualitative
Europe	1	Europe for All	2007b	Tourism	Tourism providers report on Europe for all self-assessment questionnaires: For owners or managers of hotels and self-catering establishments and Europe for all photo and measurement guides	Qualitative
United States of America	1	Van Horn	2007	Travel	Disability travel in the United States: Recent research and findings	Qualitative
Australia	2	Darcy & Dickson	2009	Tourism	A whole-of-life approach to tourism: The case for accessible tourism experiences	Qualitative
Italy	1	Rains	2009a	Tourism	Inclusive tourism – Participant or observer – Notes on the global paradigm shift towards solutions. Paper presented at the Neurology of the Third Millenium: For Well-Being in Disability	Qualitative
Italy	1	Rains	2009b	Tourism	Second policy roundtable – Accessible tourism for well-being in disability. Paper presented at the Conference as Proceedings of Neurology of the Third Millenium: For well-being in disability	Qualitative
General	2	Buhalis & Darcy	2010	Tourism	Accessible tourism concepts and issues	Mixed Methods
Australia	2	Small & Darcy	2010	Tourism	Tourism, disability and mobility	Mixed Methods
General	3	Darcy, Cameron & Pegg	2010	Tourism	Accessible tourism and sustainability: A discussion and case study	Qualitative
General	2	Buhalis & Darcy	2011	Tourism	Accessible tourism: concepts and issues, aspects of tourism	Mixed Methods
General	2	Darcy & Buhalis	2011	Tourism	Conceptualising disability: Medical, social, WHO ICF, dimensions and levels of support needs	Qualitative
General	3	Poria, Reichel & Brandt	2011	Hotel	Dimensions of hotel experience of people with disabilities: An exploratory study	Qualitative
General	5	Poria, Reichel, Brandt, Buhalis & Darcy	2011	Tourism	Blind people’s tourism experiences: An exploratory study	Qualitative
General	3	Lee, Agarwal & Kim	2012	Travel	Influences of travel constraints on the people with disabilities’ intention to travel: An application of Seligman’s helplessness theory	Quantitative
General	3	Buhalis, Darcy & Ambrose	2012	Tourism	Best practice in accessible tourism: Inclusion, disability, ageing population and tourism	Mixed Methods
Europe	n/a	Euromontana Communication Tourism report	2014	Tourism	Accessible tourism in Europe: An interesting economic market, including mountain destinations	Qualitative
Europe	n/a	European Commission, Final Report (204/PP/ENT/PPA/12/6471).	2014	Tourism	Mapping skills and training needs to improve accessibility in tourism services	Qualitative
Greece	5	Naniopoulos, Tsalis, Papanikolaou, Kalliagra & Kourmpeti	2015	Tourism	Accessibility improvement interventions realised in Byzantine monuments of Thessaloniki, Greece	Qualitative
Greece	2	Naniopoulos & Tsalis	2015	Tourism	A methodology for facing the accessibility of monuments was developed and realised in Thessaloniki, Greece	Qualitative
General	2	Loi & Kong	2015	Tourism	People with disabilities in the tourism industry: concepts and issues	Qualitative
Australia	5	Pavkovic, Lawrie, Farrell, Huuskes & Ryan	2017	Tourism	Inclusive tourism: Economic opportunities	Qualitative
Australia	2	Mckercher & Darcy	2018	Travel	Reconceptualising barriers to travel by people with disabilities.	Qualitative

**Total**	**63**	-	-	-	-	-

n/a, not applicable.

Table 9 indicates that 50% of the tourism research and disability followed a general Eurocentric perspective. In addition, Italy and Greece h ave a combined a contribution of 25% to the above mentioned theme. Australia (Oceania) has 15.6%, while the American contributed 9.4% (including Brazil and Canada as case studies) to tourism and disability knowledge.

Table 9 shows a trend towards co-authoring, which upholds collaborations and diversified views on tourism and disability inclusion. Thus, studies conducted by more than two researchers constituted 59.5% against single authorship at 31.3%. However, 9.4% of the extant literature does not indicate who the authors are. Table 9 shows authors with a single research interest as composing 28.0% of the extant literature instead of 72.0% of researchers who have repeating research interest in this regard. The authors with recurring research interests in tourism and disability include researchers such as Buhalis, Darcy and Rains. An interesting aspect here is that the mentioned authors furthered tourism and disability studies in a way that encourages in-depth research to build knowledge and understanding of disability. Thus, exploratory research on the needs and motivations of mobile-impaired persons was undertaken in 2003 (see Table 9). The said study probed for a more extensive investigation to uncover the magnitude of disability beyond physical built environmental access (see Table 9). Table 9 reflects a change of focus from the consumer to the non-disabled and disabled staff to relate disability and work-production within the tourism and hospitality industry. Case studies were used to bring a progressive narrative about the inclusion of disabled people in tourism (see Table 9). The existing research has combined disability and tourism, leaving impairments or health conditions related to tourism because disabled people are heterogeneous, just like their nondisabled counterparts.

## Discussion, contribution, implications and recommendations

The study reflects learning that the composite concept is still scarce, and the extant literature is skewed towards exploring the said concept within countries in the Global South, especially in South Africa. This seems inadequate to inform the tourism and hospitality co-created economy to understand and interpret disability and impairment related to production within a demand, supply and skills development perspective. Darcy (2010) and Darcy et al. (2020) have similar views and encourage in-depth research on different impairments in relation to tourism and disability inclusion. It is clear from the findings that the composite concept (disability, tourism and impairments or health conditions) has not yet been regarded as one of the main themes of tourism, hospitality, recreation and leisure in the academic journals and scholarly knowledge development space, especially from a country-contextualised course between 1990 and 2018. Therefore, this implies that in all identified themes such as tourism demand, supply, skills or human resources development (education), technological development and general tourism spaces, there is a need for collaborative research partnerships between tourism role players, organisations for disabled people and researchers for a contextual understanding of universal accessibility and reasonable accommodation (disability inclusion).

Based on the results, it is clear that the article can contribute by identifying gaps, though in an abstract form. The research community can regard the gaps as part of the research agenda for future studies. Accordingly, the findings reflect a need to develop knowledge on:

- characteristics of disabled tourists (different types of impairments or health conditions) using age, gender, economic participation, consumer and/or buying behaviour and those who experience disability (including the elderly)
- travel behaviours of disabled tourists
- contextual relevance of universal accessibility in tourism sectors beyond the built environment, such as attitudes, diversity management and organisational culture
- competencies (informal and formal or curriculum-based skills development) for sustainable know-how to manage and/or handle disabled people in tourism sectors
- definitions of disability inclusion and reasonable accommodation for participation in tourism and the hospitality value chain
- geospatiality and movement of disabled people (tourism sources and receiving regions) for destination management and competitiveness within accessible or inclusive tourism
- the economic value of disability inclusion, whilst disaggregating such by age, type of impairments and access needs amongst other ‘special requests’ set by tourists who participated in tourism, thereby making better market segmentation within the tourism supply and demand value chain
- interpretation of disability legislative frameworks for tourism and hospitality practices and contextualised inclusive socio-economic development (community-based development included)
- tourism and hospitality co-created innovation induced by disability inclusion (tourism for all)
- human resources policy and practices to acknowledge disabled people as potential sources of labour for employment (selection, recruitment, retention, talent and diversity management)
- employment and employability of disabled people in tourism and hospitality co-created economy, including work readiness, inclusion in the workspace, production and productivity and an inclusive labour market
- tourism and hospitality-inclusive market research and marketing communication
- inclusive tourism and hospitality management (practices, principles, planning, budgeting, economic empowerment index, information technological advancement and usage of such data for social and economic innovation)
- readiness of the tourism and hospitality co-created economic development for accessible tourism and how the multifaceted industry is prepared for the evolving concept of ‘tourism for all’.

The postulated research fields for the composite concept imply that the authors recommend the different organisations for disabled people to be open to dialogues with tourism roleplayers (education, industry and community) to bring a clear interpretation of inclusion in the lens of socio-economic production, co-creation and types of impairments and/or health conditions without compromising heterogeneity or their beneficiaries. For example, South Africa has the South Africa Disability Alliance, composed of 15 organisational members with databases that can foster the continuation of research to bridge the gaps identified in this current research. The above recommendation shares similarities with Darcy et al. (2020), Luiza (2010) and UNWTO (2020), as they believe accessible tourism is critical as one of the avenues to tourism recovery amidst and after the COVID-19 pandemic.

The study reflects potential benefits and strengths to uphold community cohesion if inclusive or accessible tourism is built on a composite concept. Research would develop sustainable fundamentals to enable tourism skills development to mainstream disability proficiency as part of a curriculum outcome. Research that upholds impact assessment can fulfil a need for knowledge, willingness and buy-in amongst internal and external tourism and hospitality stakeholders to appreciate disabled tourists beyond a niche market. The abovementioned factors implicate investment decisions and business strategies within the tourism-disability space.

## Conclusion

This study concludes that the extant literature explored disability and tourism perspectives that are inadequate to make the tourism and hospitality co-created economy understand disability inclusion in the context of tourism growth. Impairments or health conditions were not part of the existing discourse between 1990–2018. Therefore, there is a need for in-depth research to enable skills development to embed disability inclusion from a composite concept. Learnings were determined from gaps in extant literature within tourism supply, demand, technological influence, skills development and general tourism sectors. Nonetheless, research attention differs amongst researchers and the context of the countries and continents. Overall, the tripod concept of tourism, impairments (health conditions) and disability is still under-researched. One can say it is still at the infant stage, as existing knowledge is still too generic and has only ‘scraped the surface’ issues of disability, thereby leaving tourism stakeholders in a dilemma when intending to implement inclusion. Yet research is an imperative for an evidence-based argument towards systemic inclusive or accessible tourism, particularly within the areas identified by the study, namely demand, supply, skills development, technological influence and general tourism sector knowledge. Research is needed in the areas mentioned above to fill the gaps whilst liaising and engaging disability-concerned organisations. This article contributes to mapping a field of research, synthesising the state of knowledge and creating an agenda for further research whilst providing the historical overview and timeline-based milestones in research on impairments, disability and tourism as a consolidated concept. Therefore, the limitation of the study is centered on lacking an empirical approach that would augment the review of extant literature for a limited period (1990–2018) only.
